# Supplementary material for: Pharmacokinetics and Endocrine Effects of an Oral Dose of D-Pinitol in Human Fasting Healthy Volunteers
Source: Nutrients. 2022 Oct 1;14(19):4094. doi: 10.3390/nu14194094 (PMC9572189; doi:10.3390/nu14194094)
Supplement: Supplementary file 1 [file nutrients-14-04094-s001.zip › nutrients-1833620-supplementary.pdf]

Supplementary Material

Supplementary Figures

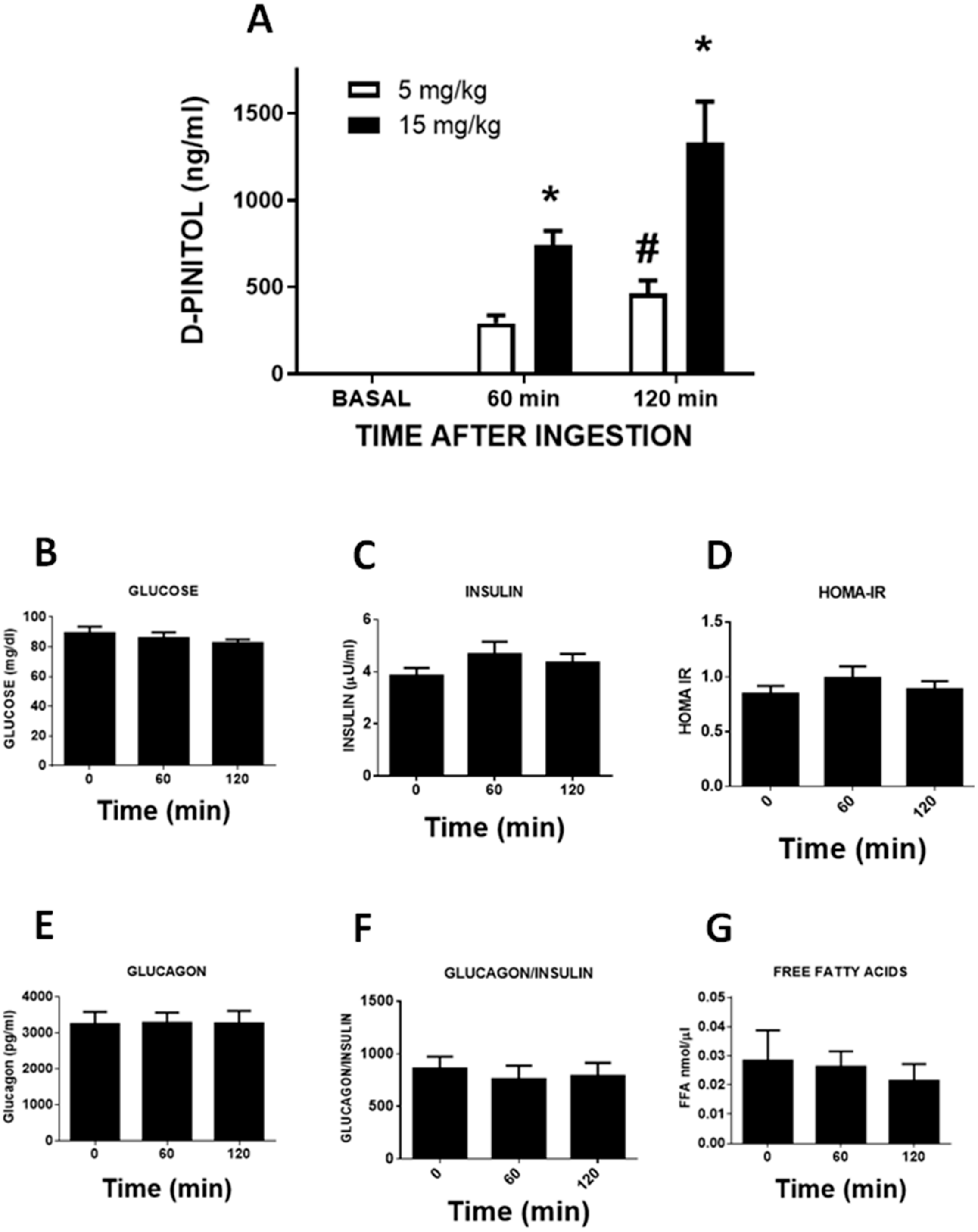

Figure S1. Lack of changes in glucose homeostasis-associated plasma parameters in fasting human subjects receiving a single oral dose of D-Pinitol (5 mg/kg). The values are means ± SEM of 6-7 measures

per group and time point. **A**. Comparative plasma D-Pinitol levels after administration of either 5 or 15 mg/kg bw dose **B**. Plasma glucose, **C.** Plasma Insulin, **D**. Insulin Resistance Index (HOMA-IR), **E**. Plasma glucagon, **F**. Plasma glucagon/Insulin ratio,**G**. Plasma free fatty acids. Differences between groups were evaluated using one-way Anova + Fisher's LSD test. * Different versus 5 mg/kg dose. #, different versus 60 min sampling time. $P<0.05$

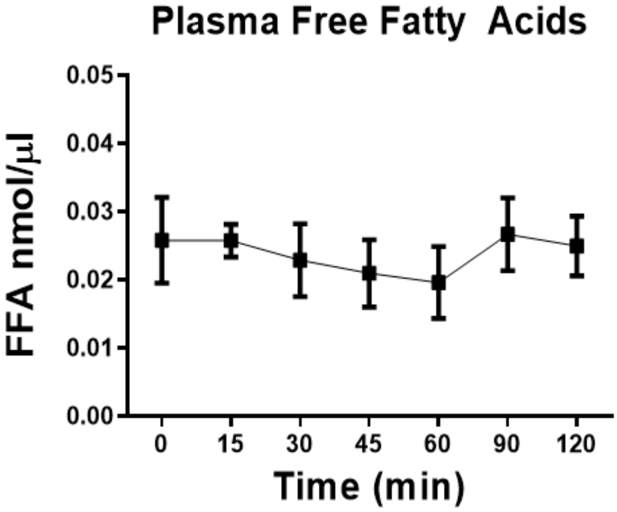

Figure S2. Plama levels of free fatty acids after a single dose of D-Pinitol in healthy human volunteers. Differences between groups were evaluated using one-way Anova + Fisher's LSD tes.
